# Online Health Information Seeking, eHealth Literacy, and Health Behaviors Among Chinese Internet Users: Cross-Sectional Survey Study

**DOI:** 10.2196/54135

**Published:** 2024-10-18

**Authors:** Diyi Liu, Shuhang Yang, Calvin Yixiang Cheng, Lin Cai, Jing Su

**Affiliations:** 1 Oxford Internet Institute, University of Oxford Oxford United Kingdom; 2 Chinese Academy of Cyberspace Studies Beijing China; 3 School of Humanities Tsinghua University Beijing China

**Keywords:** eHealth literacy, online health information seeking, health behavior, perceived information quality, health promotion, China, mobile phone

## Abstract

**Background:**

The internet has become an increasingly vital platform for health-related information, especially in upper-middle-income countries such as China. While previous research has suggested that online health information seeking (OHIS) can significantly impact individuals’ engagement in health behaviors, most research focused on patient-centered health communication.

**Objective:**

This study aims to examine how OHIS influences health behavior engagement among Chinese internet users, focusing on the role of eHealth literacy and perceived information quality in influencing relationships.

**Methods:**

An online cross-sectional survey was conducted in November 2021 among 10,000 Chinese internet users, using quota sampling based on sex, age, and urban and rural residence, in line with the 48th Statistical Report on Internet Development of China. Nonparametric tests were used to examine the differences in eHealth literacy across sociodemographic groups. Partial correlation analysis and stepwise linear regression were conducted to test the associations between key variables. Confirmatory factor analysis and structural equation modeling were conducted to test the hypotheses.

**Results:**

Our study identified significant disparities in functional and critical eHealth literacy between urban and rural residents across age groups, income levels, education backgrounds, and health conditions (all *P*<.001). In terms of sex and regional differences, we found higher functional literacy among female users than male users, and critical literacy varied significantly across different regions. The proposed structural model showed excellent fit (*χ*^2^_404_*=*4183.6, *χ*^2^_404_=10.4*,* *P*<.001; root mean square error of approximation value of 0.031, 95% CI 0.030-.031; standardized root mean square residual value of 0.029; and comparative fit index value of 0.955), highlighting reciprocal associations between 2 types of eHealth literacy and OHIS. Participants’ functional eHealth literacy, critical eHealth literacy, and OHIS have positive impacts on their health behavioral engagement. Perceived information quality was found to mediate the influence of OHIS on health behavior (b=0.003, 95% CI 0.002-0.003; *P*<.001).

**Conclusions:**

The study revealed the pathways linking sociodemographic factors, eHealth literacy, OHIS, and perceived information quality and how they together influenced health outcomes. The findings underscore the significance of enhancing eHealth literacy and improving information quality to promote better health outcomes among Chinese internet users.

## Introduction

### Background

The internet has become crucial for health information dissemination in the digital era [1,2]. Online health information seeking (OHIS) has gained popularity due to its accessibility, wide information coverage, ease of use, affordability, and anonymity [3,4], especially in upper-middle-income countries such as China, where health care resources and in-person medical appointments are limited [5,6]. As of December 2022, China’s internet penetration rate has reached 75.6%, with 1.067 billion internet users accessing health information on the web [7]. The shift to digital health is essential for improving health knowledge, increasing confidence in managing health issues, and promoting healthy behaviors [8,9]. Nevertheless, previous research has noted that information alone might not be sufficient to affect optimal health-related well-being [10]. The notion of health literacy, first introduced in China in 2005 and widely acknowledged as a cost-effective measure for improving public health [11], has evolved into eHealth literacy, which is a foundational skill set that underpins the use of information and communication technologies for health [12-14]. While research has examined digital health literacy and health information seeking among specific user segments within China, such as community-dwelling older population [9], rural residents [15], or active social media users [16], and some studies have leveraged nationwide survey samples [6,17], a comprehensive analysis of the broader population’s engagement with online health resources is needed.

### OHIS and Health Behaviors

Health behavior, distinct from medical treatment, concerns how health interventions and societal norms affect the health of individuals’ lifestyles [18]. While previous research has extensively focused on information seeking and literacy in patient-centered health communication [19,20], OHIS is not limited to individuals facing health threats [1]. More commonly, the general public turns to the internet to find information on leading healthier lifestyles, which warrants examination into how the wider population uses the internet for health-related decisions [21]. By providing access to more health-relevant information, OHIS might enable a more accurate assessment of health status, disease outbreak severity, and the need for health protection measures [8]. It could contribute to individuals’ perceived control over health threats and reduce negative emotions associated with uncertainty [22]. As most existing literature has acknowledged the potential of OHIS to contribute to better health outcomes, we first examine the direct path.

- Hypothesis 1: OHIS is positively associated with individuals’ engagement in health behaviors.

### Linking eHealth Literacy With OHIS and Health Behaviors

Among existing scholarship on internet-facilitated health communication and promotion, the integrative model of eHealth use underscores that macrolevel social disparities, often known as the digital divide in the realm of health communication [23,24], manifest as microlevel individual variations in the orientation and efficacy of OHIS, influencing people’s internet use for health-related purposes and ultimately their health outcomes [25]. For instance, previous research found that female, older, married, and better-educated Chinese internet users engage in OHIS more frequently [6] and that people’s health status could alter their health behavioral engagement [26].

As the integrative model of eHealth use suggests, online health resources can only improve health outcomes if the public has adequate eHealth literacy and avoids low-quality and harmful content [27]. eHealth literacy refers to a person’s perceived ability to (1) have access to health information on the web and (2) understand the health information accessed [13]. It not only affects individuals’ OHIS behaviors [28-30] but also directly and indirectly engages with sequential health outcomes [26,31]. Centering on the cognitive mechanisms linking OHIS and health behaviors, the study delved into how 2 interrelated dimensions of transactional eHealth literacy influence the process. *Functional eHealth literacy* refers to individual users’ perceived ability to acquire online health information, while *critical eHealth literacy* involves more advanced cognitive processes related to information appraisal, including evaluating the reliability, validity, credibility, and applicability of health information [32]. We first examine the impact of Chinese internet users’ sociodemographic factors and health status on their eHealth literacy.

- Research question 1: How are Chinese netizens’ sociodemographic factors and health status associated with their (1) functional and (2) critical eHealth literacy, respectively?

Higher eHealth literacy might encourage users to resort to the internet, leading to a stronger likelihood of OHIS under health motivation [25,33]. With better functional and critical literacy, individuals would have more ability to access health information and make efficient use of online searching tools and technological devices [28,34]. Conversely, people with less knowledge and confidence in using digital devices are found less likely to conduct OHIS [35,36]. While an association between eHealth literacy and actual eHealth use is recognized, previous research has lent mixed support for the direction of the relationship, and we cannot rule out the possibility of a bidirectional association. Moreover, individuals’ personal traits may be associated with both eHealth literacy as well as OHIS [26,37], thus further confounding the process. In this study, we deployed structural equation modeling (SEM) to assess the possible bidirectional relationship between the 2 types of eHealth literacy and OHIS. High critical literacy might contribute to better well-being and engagement in healthy behaviors [38,39], as it can increase levels of self-engagement, initiative, and control over health concerns and self-care management [19]. Therefore, we propose 2 hypotheses.

- Hypothesis 2: Chinese internet users’ (1) functional and (2) critical eHealth literacy have reciprocal associations with their OHIS.
- Hypothesis 3: Chinese internet users’ (1) functional and (2) critical eHealth literacy are positively associated with their engagement in health behaviors.

### Mediator: Perceived Quality of Online Health Information

Apart from eHealth literacy, users’ evaluation and satisfaction with obtained health information or experiences using different sources play an important role in health outcomes [40]. Extant theoretical models suggest that the effectiveness of interactive media use for health information is conditioned on users’ information processing while engaged with the media and content, which may or may not lead to optimal health outcomes. For instance, the 3-stage model of health promotion using interactive technology proposed that the use of interactive online health resources functions through the interplay of the characteristics of user, media, and health message, potentially contributing to users’ health maintenance and improvement [41,42]. Our study specifically focuses on users’ attitudes toward the quality of health sources and the information itself [43], as a previous study denoted that content-related indicators and criteria were used the most in credibility evaluation across different information sources compared with the other functionalities [44]. Specifically, we measure perceptions of online health information in terms of scientific rigor, timeliness, accuracy, objectiveness, credibility, applicability, as well as potential harm. We propose that the psychological mechanism underlying health information processing is contingent on individuals’ evaluation of the quality of online health information.

- Hypothesis 4: Chinese internet users’ perceived quality of online health information will mediate the relationship between OHIS and health behavioral engagement.

### Objectives

This study aimed to examine the underlying mechanism by which OHIS influences the engagement in health behaviors of Chinese internet users across various sociodemographic groups. Our conceptual framework proposes a structural model highlighting the interrelationships among users’ OHIS behavior, their self-assessed ability to acquire information on the web (functional literacy), and their ability to critically appraise acquired health information (critical literacy), which potentially affect their health outcomes. In addition, the study accounts for the mediating effect of users’ perceptions of online health information quality on the pathways.

## Methods

### Study Design

A cross-sectional online survey was conducted from November 1, 2021, to November 26, 2021, among Chinese internet users accessing health information on the web. The reporting follows the Checklist for Reporting Results of Internet e-Surveys (CHERRIES; Multimedia Appendix 1 [45,46]).

Our questionnaire was developed with input from media, communication, and public health experts to ensure content relevance and clarity. A group of subject matter experts reviewed the original questionnaire and the adapted scales for eHealth literacy and health behavior. The sample size was determined to balance statistical power with budgetary constraints. We targeted a 95% CI with a 1% margin of error for China’s internet population (1.011 billion) as of June 2021. In addition, we followed the rule of thumb for the minimum sample size for SEM based on our proposed model [47]. We used quota sampling, considering national representativeness based on sex, age groups, as well as urban and rural residence. Quotas were determined using data from the 48th Statistical Report on Internet Development of China conducted by the China Internet Network Information Center [48]. The survey was distributed through a professional Chinese online survey platform, IDiaoYan (Zhongyan Technology) [45]. We conducted a closed survey accessible exclusively to registered panelists.

**Figure 1:**
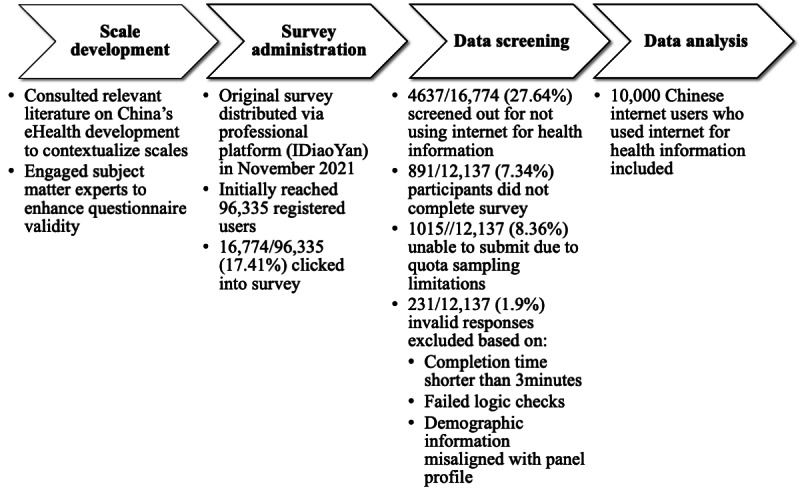
Flowchart illustrating survey development and data screening process.

Our study initially reached 96,335 registered users of the platform, among which 17.41% (n=16,774) users clicked into the first page of the survey. In a prescreening question, we asked whether respondents used the internet for health information, and 27.64% (4637/16,774) of the participants who answered “no” to this question were screened out. Of the 12,137 remaining responses, 7.34% (n=891) were incomplete and 8.36% (n=1015) participants were unable to submit responses due to quota sampling limitations, where response collection ceases once the quota for a specific group is reached. We also excluded invalid responses (231/12,137, 1.9%) based on the following criteria: (1) completion time <3 minutes, (2) failed logic checks, or (3) filled-in demographic information misaligned with the registered profile in the panel. Consequently, the final sample included 10,000 complete and valid responses. The survey development is illustrated in the flowchart of Figure 1, and the questionnaire in English and original language (Chinese) are included in Multimedia Appendix 2.

### Ethical Considerations

The research protocol for this study was reviewed and approved by the School of Humanities of Tsinghua University and Chinese Academy of Cyberspace Studies. At the beginning of the survey, participants were presented with information about the purpose and procedures of the study, as well as how the data would be handled. They were only allowed to proceed with the study after reading the information and providing informed consent. For participants aged <18 years, parents or guardians provided consent on their behalf, and these participants were required to complete the survey under parental guidance. Participation was on a voluntary basis. All collected data were anonymized, removing any identifiers that could directly or indirectly link the data to individual participants. The data collection and storage protocols were in full compliance with the Personal Information Protection Law of China. Respondents who submitted valid responses were rewarded with 5000 bonus points, equivalent to CNY 5 (US $0.8) through the survey platform’s loyalty points program.

### Measurements

#### Frequency of OHIS

**Figure 2:**
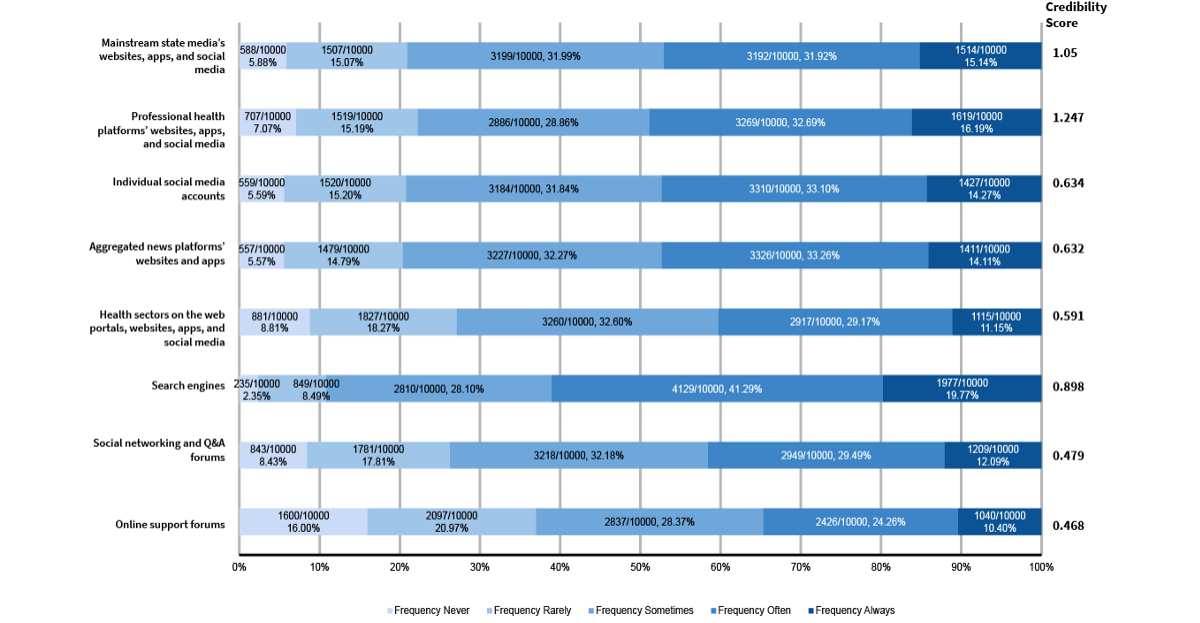
Frequency of use and perceived credibility of different health information channels (N=10,000).

Drawing on previous literature [41,49], we operationalized individuals’ OHIS as the frequency of participants’ engagement in health information seeking through different online channels. Participants were asked to indicate how often they sought health information from (1) mainstream media, (2) professional health media, (3) aggregator news platforms, (4) web portals, (5) open forums, (6) online support forums, (7) search engines, and (8) individual social media accounts. Examples of each information source were provided, as shown in Figure 2 and Multimedia Appendix 2. Responses ranged from “1=never” to “5=always,” indicating the frequency of OHIS through each channel. Our intended latent construct of OHIS showed good reliability (Cronbach α=0.85; mean 3.30, SD 0.77).

#### Credibility of Information Source

We calculated a credibility score for each information channel by reverse coding the credibility ranks given by participants. Specifically, participants ranked their top 3 information sources in order of perceived credibility. We later assigned a weight of 3 to the most credible, 2 to the second most credible, and 1 to the third most credible source. We then averaged these weighted rankings across participants to examine the credibility score for each of the 8 sources assessed. The resulting credibility scores ranged from 0 to 3, where a higher score signified greater perceived credibility.

#### eHealth Literacy

eHealth literacy was assessed using a modified version of the eHealth Literacy scale, which had been translated into Chinese and demonstrated good reliability and validity [13,50]. Our study introduced a 2-factor model based on functional and critical literacy. Specifically, we assessed *functional literacy* by asking participants to what extent they were capable of obtaining and accessing health information in the Web 2.0 context [13,21]. Sample items include “I know what health resources are available on the Internet” and “I am capable of using mobile devices to search for health information.” *Critical literacy* focused on how participants assessed and evaluated health information obtained from the internet to make health decisions [14,51], using items such as “I can tell high-quality from low-quality health resources on the Internet.” Response options for both variables included a 5-point Likert scale ranging from “1=totally disagree” to “5=totally agree.” The scores were then averaged to generate 2 indices of users’ functional literacy (Cronbach α=0.804; mean 3.84, SD 0.63) and critical literacy (Cronbach α=0.73; mean 3.55, SD 0.68), showing good reliabilities of the adapted eHealth literacy scales.

#### Health Behavior

Our study measured health maintenance behaviors using 4 self-reported items that represent people’s commitment to reduce negative health outcomes and facilitate psychological and behavioral well-being [52]. The scale was revised based on previous literature [26]. Examples of the items include mental health management as well as good hygiene habits. Participants rated these 4 items on a 5-point scale ranging from “1=not at all” to “5=extremely well.” Cronbach α for this variable was reasonably reliable (Cronbach α=0.74; mean 3.77, SD 0.69).

#### Perceived Quality of Online Health Information

We measured participants’ perceived quality of online health information using 5 items assessing the reliability, accuracy, and applicability of health-related online content [28,43]. Respondents were asked to evaluate the quality of internet health information based on to what degree they think that online health information (1) is supported by reliable scientific evidence; (2) is credible and reliable; (3) does not pose a risk to their personal health and well-being; (4) aligns with the latest advancements and consensus within the medical science community; and (5) is actionable and applicable in real-life scenarios. Responses were scored on a 5-point scale from “1=strongly disagree” to “5=strongly agree” (Cronbach α=0.77; mean 3.51, SD 0.65).

#### Control Variables

We collected participants’ sex, age groups, education levels, monthly income, residential areas, and provinces. In addition, participants self-evaluated health status was also measured using scales validated by a previous study [17].

### Data Analysis

In our study, we first conducted univariate and multivariate analyses, partial correlation, and regression analysis using SPSS software (version 29.0; SPSS Inc). To assess the normality of eHealth literacy, OHIS, health behavior, and perceived quality, we applied the Kolmogorov-Smirnov test. Non-normal variables were reported as mean (SD) as well as median (IQR). Categorical variables were reported as frequency and percentages. Differences between sex (female and male) and residence types (rural and urban) were examined using the Mann-Whitney *U* test, while the Kruskal-Wallis test was used for comparison across various age groups, educational levels, income brackets, regions, and health status. We considered *P* values <.05 (2-sided) to be statistically significant. Partial correlations were examined among functional and critical eHealth literacy, OHIS, evaluation, and health behavior while controlling age, sex, education, income, health status, residence, and regional distribution. We then conducted stepwise linear regression with OHIS and health behavior as dependent variables, respectively, and categorical user characteristics, eHealth literacy, and perceived health information quality as independent variables. Using a stepwise method (*P*<.05 as the criterion for entry and *P*>.10 as the criterion for exclusion), all possible combinations of variables were first tested, and the best combinations were selected based on model fit and significance. In addition, we assessed multicollinearity in our regression by examining the variance inflation factor.

To test our hypotheses, we used a 2-step approach following previous research [49,53]. First, to assess the reliability and validity of the latent variables in our model, we performed a confirmatory factor analysis (CFA). This step evaluated the proposed measurement model. Given that we have a relatively large sample size (N=10,000) that strongly influences the result of the chi-square test, we did not refer to the cutoff value 3 of normalized chi-square but the other absolute and incremental model fit indices (eg, root mean square error of approximation [RMSEA], comparative fit index [CFI], and standardized root mean square residual [SRMR]). After the CFA, we applied SEM to examine the pathways among our key variables while controlling for sex, age groups, residence, educational levels, income, and health status. We used a Bootstrap analysis with a 95% CI to estimate the parameters and their associated SEs. Both the CFA and SEM analyses were conducted using the *Lavaan* package [54] in R software (version 4.2.2; R Foundation for Statistical Computing).

## Results

### Preliminary Analysis

#### Participants Characteristics and OHIS Engagement

Our study sample included 10,000 participants from 31 provinces in mainland China. The details of participants’ demographic distribution and self-reported health status are presented in Table 1. Table S1 in Multimedia Appendix 3 contains detailed descriptive statistics of eHealth literacy across groups, and Table S2 of Multimedia Appendix 3 contains provincial distribution of participants’ eHealth literacy. The sampling followed the 48th Statistical Report on Internet Development of China, where most Chinese internet users resided in urban areas (7060/10,000, 70.6%), were aged between 20 and 49 years (5640/10,000, 56.4%), and were relatively evenly distributed between female (4880/10,000, 48.8%) and male (5120/10,000, 51.2%) users. It should be noted that our respondents were mostly well educated, with about 60% holding a bachelor (3393/10,000, 33.9%) or associated degree (2588/10,000, 25.9%). Furthermore, more than half (5628/10,000, 56.3%) of the participants were from China’s eastern provinces. Users’ health status varied, with most self-reporting good health condition (3777/10,000, 37.8%) and 26.6% (2661/10,000) experiencing subhealth symptoms, such as fatigue and poor appetite. Severe health conditions, such as cancer, were relatively rare among all participants (46/10,000, 0.5%).

**Table 1:** Demographic characteristics, health status, and distribution of eHealth literacy (N=10,000).

Demographics			Participants, n (%)		Test statistics for functional literacy^a^		*P* value		Test statistics for critical literacy		*P* value
**Sex**					3.121		.002		–0.973		.33
	Female	4880 (48.8)									
	Male	5120 (51.2)									
**Age group (y)**					242.323		<.001		116.489		<.001
	<19	1560 (15.6)									
	20 to 29	1740 (17.4)									
	30 to 39	2030 (20.3)									
	40 to 49	1870 (18.7)									
	50 to 59	1590 (15.9)									
	>60	1210 (12.1)									
**Residential area**					–10.293		<.001		–3.740		<.001
	Urban	7060 (70.6)									
	Rural	2940 (29.4)									
**Education level**					192.303		<.001		62.955		<.001
	Primary school or less	174 (1.74)									
	Middle school	1066 (10.66)									
	High school or secondary vocational school	2499 (24.99)									
	Associate degree	2588 (25.88)									
	Bachelor degree	3393 (33.93)									
	Master and above	280 (2.8)									
**Income level** ^b^					128.293		<.001		226.919		<.001
	<¥1500	1267 (12.67)									
	¥1500 to 3000	1286 (12.86)									
	¥3001 to 5000	2294 (22.94)									
	¥5001 to 8000	2632 (26.32)									
	¥8001 to 12,000	1629 (16.29)									
	¥12,001 to 20,000	693 (6.93)									
	>¥20,000	199 (1.99)									
**Health status**					74.46		<.001		17.458		<.001
	Experiencing a severe disease	46 (0.46)									
	Experiencing chronic diseases	1397 (13.97)									
	Subhealth symptoms	2661 (26.61)									
	Not bad	2119 (21.19)									
	Good	3777 (37.77)									
**Region**					4.57		.03		49.240		<.001
	East	5628 (56.28)									
	Central	1998 (19.98)									
	West	1630 (16.3)									
	Northeast	744 (7.44)									

^a^Test statistics were reported for dichotomous variables (*z* score) and multicategorical variables (*χ^2^*) alongside *P* value.

^b^A currency exchanged rate of 1¥=US $0.16 is applicable.

We measured OHIS by examining the frequency of use of online sources. As shown in Figure 2, search engines, such as Baidu*,* emerged as primary tools for health-related inquiries (“often”: 4130/10,000, 41.3% and “always”: 1980/10,000, 19.8%). Conversely, emerging specialized online support forums, such as the bulletin board system forum for patients with diabetes, saw less frequent engagement, with 16% (1600/10,000) of the participants indicating that they “never” have used such platforms. Regarding source credibility, among the 8 channels assessed, professional health media outlets were perceived as the most credible. State media outlets, such as Xinhua News and People’s Daily, were also rated as relatively credible. However, social networking (question and answer) forums and online support communities were regarded as less credible.

#### Univariate Analysis of eHealth Literacy Across Different Sociodemographic Groups

Our analyses revealed differences in both functional and critical eHealth literacy across various demographic, socioeconomic, and health-related variables. The detailed descriptive statistics across groups can be found in Table S1 in Multimedia Appendix 3. The Mann-Whitney *U* test indicated that female users have a significantly higher functional literacy than their male counterparts (*z* score=3.12; *P*=.002). No significant difference was found in critical literacy (*z* score=–0.97; *P*=.33). A digital divide was observed between urban and rural residents, evident in both functional literacy (*z* score=–10.29; *P*<.001) and critical literacy (*z* score=–3.74; *P*<.001). Multicategorical group comparison showed variations in literacy levels across age cohorts in functional literacy (*χ*^2^_5_=242.3; *P*<.001) and critical literacy (*χ*^2^_5_=116.5; *P*<.001). Internet users with different income levels demonstrated variations in both functional literacy (*χ*^2^_6_=128.3; *P*<.001) and critical literacy (*χ*^2^_6_=226.9; *P*<.001). Similarly, respondents with different educational backgrounds also showed varied levels of functional literacy (*χ*^2^_5_=192.3; *P*<.001) and critical literacy (*χ*^2^_5_=62.9; *P*<.001). Furthermore, there were marked differences based on participants’ health conditions in functional literacy (*χ*^2^_4_=74.5; *P*<.001) and critical literacy (*χ*^2^_4_=17.5; *P*<.001). Notably, we did not observe significant regional differences in internet users’ functional literacy (*χ*^2^_3_=3.6; *P*=.21); however, users’ critical literacy demonstrated significant regional variations across East, Central, West, and Northeast China (*χ^2^*_3_=49.2; *P*<.001).

#### Multivariate Analysis on Social and Individual Differences of OHIS and Health Behavior

Partial correlation analysis, as detailed in Table 2, revealed significant positive associations among the 5 key variables: functional literacy, critical literacy, OHIS, perceived information quality, and health behavior, controlling for covariates (all *P*<.001).

**Table 2:** Partial correlation analysis (Pearson *r* and 2-tailed *P* value) among key variables^a^.

		Functional literacy	Critical literacy	Health behavior	Perceived quality	OHIS^b^
**Functional literacy**						
	*r*	1	0.606	0.331	0.416	0.136
	*P* value	—^c^	<.001	<.001	<.001	<.001
**Critical literacy**						
	*r*	0.606	1	0.320	0.540	0.255
	*P* value	<.001	—	<.001	<.001	<.001
**Health behavior**						
	*r*	0.331	0.320	1	0.290	0.257
	*P* value	<.001	<.001	—	<.001	<.001
**Perceived quality**						
	*r*	0.416	0.540	0.290	1	0.276
	*P* value	<.001	<.001	<.001	—	<.001
**OHIS**						
	*r*	0.136	0.255	0.257	0.276	1
	*P* value	<.001	<.001	<.001	<.001	—

^a^Partial correlation coefficients were calculated with sex, age groups, residence, region, education, income, and health status as control variables.

^b^OHIS: online health information seeking.

^c^Not applicable.

The results of stepwise linear regression investigated the associations between user-oriented characteristics and their OHIS and health behavioral engagement. Users’ sex and regional differences were excluded in the final model predicting OHIS (Table 3), while users’ residence types and regional differences were excluded in the final model predicting health behavior (Table 4).

Aside from the excluded factors, all considered sociodemographic factors, eHealth literacy, and perceived information quality showed statistically significant main effects on both OHIS and health behaviors (all *P*<.001). Furthermore, the variance inflation factor values were <2 across the model, suggesting no collinearity issues and thereby affirming the reliability of the regression analyses.

**Table 3:** Stepwise linear regression predicting online health information seeking (OHIS)^a^.

Included predictors	b^b^ (SE)	β	*t* test (*df*)	*P* value	VIF^c^
Income	0.10 (0.01)	.20	17.38 (9991)	<.001	1.60
Age	0.04 (0.01)	.07	6.13 (9991)	<.001	1.73
Health condition	–0.04 (0.01)	–.06	–6.11 (9991)	<.001	1.35
Residence	0.11 (0.02)	.06	5.97 (9991)	<.001	1.35
Education level	0.05 (0.01)	.07	5.85 (9991)	<.001	1.73
Functional literacy	–0.07 (0.01)	–.06	–4.86 (9991)	<.001	1.66
Critical literacy	0.20 (0.01)	.17	13.85 (9991)	<.001	1.92
Perceived quality	0.23 (0.01)	.20	17.75 (9991)	<.001	1.51

^a^The result shows the final model in stepwise regression predicting OHIS: *R*^2^=0.187, adjusted *R*^2^=0.186, *F*_1,9991_=315.21 (*P*<.001). Sex and regional distribution were excluded.

^b^b: unstandardized coefficient.

^c^VIF: variance inflation factor.

**Table 4:** Stepwise linear regression predicting health behavior^a^.

Included predictors	b^b^ (SE)	β	*t* test (*df*)	*P* value	VIF^c^
Income	0.03 (0.01)	.06	5.62 (9991)	<.001	1.54
Age	0.06 (0.01)	.15	12.75 (9991)	<.001	1.71
Education level	0.04 (0.01)	.07	6.07 (9991)	<.001	1.62
Health condition	0.05 (0.01)	.08	7.22 (9991)	<.001	1.35
Sex	0.06 (0.01)	.05	5.08 (9991)	<.001	1.03
Functional literacy	0.22 (0.01)	.20	16.84 (9991)	<.001	1.66
Critical literacy	0.12 (0.01)	.12	9.81 (9991)	<.001	1.92
Perceived quality	0.15 (0.01)	.14	12.69 (9991)	<.001	1.51

^a^The result shows the final model in stepwise regression predicting health behavior: *R*^2^=0.187, adjusted *R*^2^=0.187, *F*_1,9991_=160.99 (*P*<.001). Residence and regional distribution were excluded.

^b^b: unstandardized coefficient.

^c^VIF: variance inflation factor.

### Hypothesis Testing

Our research model posited that participants’ functional and critical eHealth levels exhibited reciprocal relationships with OHIS. This, in turn, was posited to enhance their perceptions of the quality of online health information, thereby increasing engagement in health-promoting behaviors. The model mapped all direct and indirect pathways linking 5 key variables, with the literacy-OHIS pathways modeled as bidirectional. We also estimated the covariance between these 5 latent variables in our model, considering their correlations. To control for confounding effects, we included sex, age group, residential area, educational background, income, and health status as covariates in our structural model. Notably, based on the results of stepwise linear regression analyses, internet users’ regional difference was not incorporated as a covariate in the final model.

### Measurement Model

Above all, our measurement model (Table 5) demonstrated excellent model fit (*χ^2^*_289_*=*2889.03*, χ^2^/df*=10, *P*<.001; RMSEA=0.030, 95% CI 0.029-0.031; SRMR=0.029; CFI=0.968), underscoring the structural integrity of the 5 key constructs. One item from OHIS (ie, seeking information via search engine) was dropped due to low factor loading. All remaining factor loadings were above the recommended threshold of 0.5, showing acceptable indicator reliability. The constructs also exhibited satisfactory content reliability, with Cronbach α coefficients ranging from 0.73 to 0.86 and composite reliability values spanning 0.73 to 0.86. While our average variance extracted (AVE) fell slightly below the standard 0.5 threshold, Fornell and Larcker [55] have discussed that given that AVE is a more stringent measurement, researchers might still conclude the establishment of convergent validity with satisfactory composite reliability. This notion is particularly relevant to our study’s tailored adaptation of existing scales. Given these considerations and supported by supplementary research [56], we concluded that the measurement model has established convergent validity based on (1) AVE values marginally <0.5; (2) factor loadings all >0.5, showing strong item-to-construct relationships; and (3) composite reliability values >0.7 across all constructs, and thus collectively affirming the convergent validity of the model.

**Table 5:** Statistical outcomes of confirmatory factor analysis^a^.

Constructs			Standardized factor loading		*z* score		AVE^b^			CR^c^	
**OHIS^d^ (Cronbach α=0.86)**								0.467			0.86
	C1: mainstream media	0.674		—^e^							
	C2: professional health media	0.663		54.046							
	C3: aggregator platforms	0.647		59.950							
	C4: web portals	0.779		65.443							
	C5: open Q & A^f^ forums	0.666		58.870							
	C6: online support forums	0.757		63.242							
	C7: individual social media accounts	0.542		46.222							
**Functional literacy (Cronbach α=0.80)**								0.406			0.80
	FL1: I know how to find helpful health resources on the internet.	0.651		—							
	FL2: I know what health resources are available on the internet.	0.633		51.207							
	FL3: I am capable of using mobile devices (eg, smartphones and tablets) to search for health information online.	0.635		50.271							
	FL4: I can effectively use relevant keywords and logical search operators when querying or retrieving health information online.	0.615		49.369							
	FL5: I have the skills to open and navigate different web pages and websites to access health information across the internet.	0.652		49.608							
	FL6: I know how to bookmark or save useful health information from online sources.	0.634		49.341							
**Critical literacy (Cronbach** **α=0.73)**								0.398			0.73
	CL1: I have the skills I need to evaluate the health resources I find on the internet.	0.621		—							
	CL2: I can tell high quality from low-quality health resources on the internet.	0.622		46.044							
	CL3: I can distinguish between different sources of health information, such as authoritative sources and primary sources (eg, medical records).	0.636		49.964							
	CL4: I feel confident in using information from the internet to make health decisions.	0.642		45.410							
**Perceived quality (Cronbach** **α=0.77)**								0.401			0.77
	E1: The content of online health information is supported by reliable scientific evidence.	0.666		—							
	E2: The sources of online health information are credible and reliable.	0.676		52.905							
	E3: Accessing online health information does not pose a risk to my personal health and well-being.	0.583		45.871							
	E4: Online health information aligns with the latest advancements and consensus within the medical science community.	0.621		50.624							
	E5: The advice contained in online health information is actionable and applicable in real-life scenarios.	0.624		49.412							
**Health behaviors (Cronbach** **α=0.74)**								0.421			0.74
	HB1: dietary balance	0.685		—							
	HB2: active exercise	0.664		50.063							
	HB3: mental health maintenance	0.656		44.445							
	HB4: hygiene behavior	0.584		43.064							

^a^Model fit: *χ*^2^_289_*=*2889.03, *χ*^2^*/*289=10, *P*<.001; root mean square error of approximation of 0.030; standardized root mean square residual of 0.029; comparative fit index value of 0.968.

^b^AVE: average variance extracted.

^c^CR: composite reliability.

^d^OHIS: online health information seeking.

^e^Items constrained for identification purposes.

^f^Q and A: question and answer.

### Structural Model

**Figure 3:**
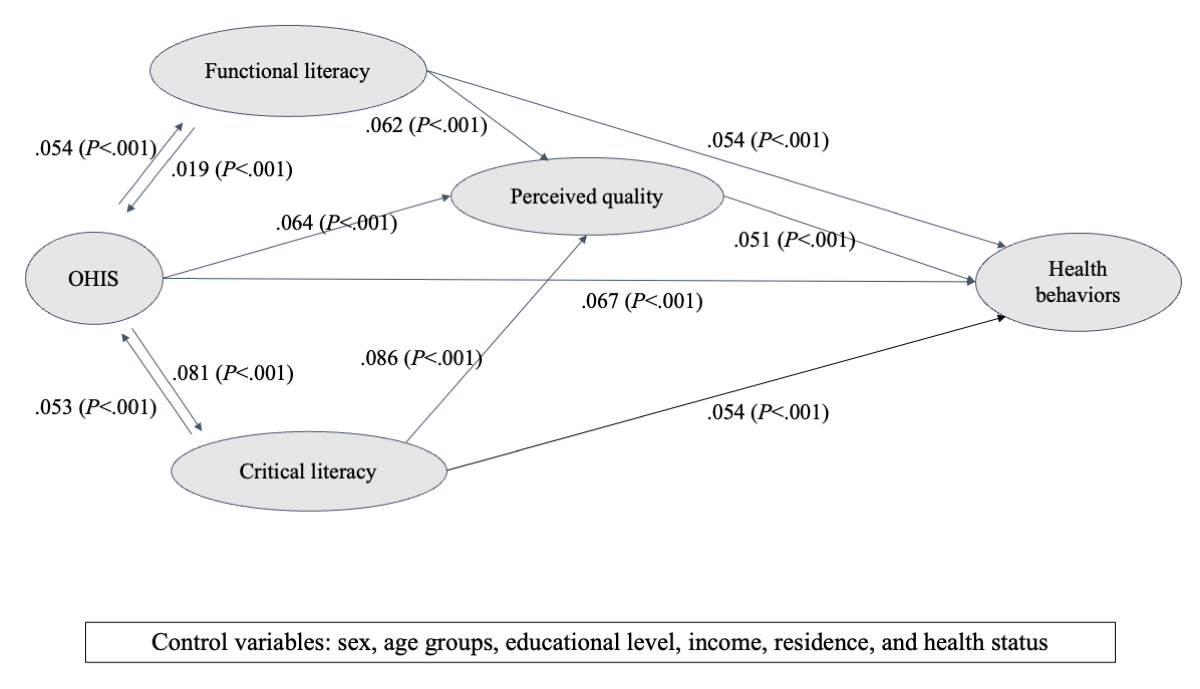
Path analysis results of structural equation model. Arrows depict the direction of effect. Reported values are standardized coefficients (β). OHIS: online health information seeking.

The result of the SEM is presented in Figure 3 and Table 6 (both unstandardized and standardized coefficients are reported). Several model fit measures suggest that our final model was found to be a good fit to the data with *χ^2^*_404_*=*4183.6, *χ2/df=*10.36 (*P*<.001), RMSEA=0.031 (95% CI 0.030-0.031), SRMR=0.029, and CFI=0.955.

**Table 6:** Hypotheses testing for direct effects^a^.

Hypothesized direct paths		b^b^ (SE)	*P* value	β	Results
Hypothesis 1: OHIS^c^ to health behavior		0.057 (0.003)	<.001	.067	√
**Hypothesis 2a**					
	Functional literacy to OHIS	0.025 (0.002)	<.001	.019	√
	OHIS to functional literacy	0.042 (0.003)	<.001	.054	√
**Hypothesis 2b**					
	Critical literacy to OHIS	0.068 (0.003)	<.001	.053	√
	OHIS to critical literacy	0.063 (0.003)	<.001	.081	√
Hypothesis 3a: functional literacy to health behavior		0.060 (0.003)	<.001	.054	√
Hypothesis 3b: critical literacy to health behavior		0.059 (0.003)	<.001	.054	√
Hypothesis 4a: OHIS to perceived quality		0.051 (0.003)	<.001	.064	√
Hypothesis 4b: perceived quality to health behavior		0.054 (0.002)	<.001	.051	√

^a^*χ*^2^_404_*=*4183.6, *χ*^2^*/*404*=*10.36 (*P*<.001), root mean square error of approximation of 0.031 (95% CI 0.030-0.031), standardized root mean square residual of 0.029, and comparative fit index value of 0.955.

^b^b=unstandardized coefficient. Bootstrap sample=5000 with replacement.

^c^OHIS: online health information seeking.

Specifically, for hypothesis testing, our result showed that OHIS is positively associated with individuals’ engagement in health behaviors (b=0.057, SE 0.003, *P*<.001, β=.067). Hypothesis 2 aims to test the relationships between functional literacy, critical eHealth literacy, and OHIS. Considering the statistical significance and relative magnitudes of the standardized regression coefficients of the 4 paths, we conclude that there are bidirectional relationships between functional or critical eHealth literacy and OHIS. Specifically, participants’ functional literacy positively predicts the frequency of OHIS (b=0.025, SE 0.002, *P*<.001, β=.019), while the opposite path is also significant (b=0.042, SE 0.003, *P*<.001, β=.054). Furthermore, respondents’ critical literacy positively predicts their OHIS (b=0.068, SE 0.003, *P*<.001, β=.053), while more frequent information seeking also has a significant effect on critical literacy (b=0.063, SE 0.004, *P*<.001, β=.081). Hypothesis 2 was supported.

Hypothesis 3 tackles the direct relationships between both functional and critical eHealth literacy and health behaviors. Results showed that internet users’ functional eHealth literacy is positively associated with their engagement in healthier behaviors (b=0.060, SE 0.003, *P*<.001, β=.054). Critical eHealth literacy also positively predicts health behavioral engagement (b=0.059, SE 0.003, *P*<.001, β=.054). Hypotheses 3a and 3b were supported. OHIS is associated with individuals’ evaluation of online health information quality (b=0.051, SE 0.003, *P*<.001, β=.064), and the direct path between perceived information quality and health behaviors is also significantly positive (b=0.054, SE 0.002, *P*<.001, β=.051). To further examine the mediating effect of perceived information quality between OHIS and health outcomes, an unstandardized indirect effect was computed for each of the 5000 bootstrapped samples as well as the 95% CI. The indirect effect was statistically significant that users’ evaluation of online information positively mediates the relationship between OHIS and health behaviors (b=0.003, 95% CI 0.002-0.003; *P*<.001). This lent support to the mediating effect of perceived OHIS quality, and thus, we accepted hypothesis 4.

## Discussion

### Principal Findings

While previous studies have identified various factors that are associated with OHIS or health behavioral intentions in the Chinese context [6,12,16,17], few have illustrated the full pathways from development to application of individual eHealth literacy, information seeking, and subsequent health wellness. Building upon existing theoretical models of eHealth use for health promotion [25,42], this paper examined how Chinese internet users’ perceptual abilities in leveraging eHealth information were constituted within macrolevel socioeconomic structures and influenced by individual-level health concerns. We found that eHealth literacy interrelates with use of online health information and ultimately affects users’ health behavioral engagement. Furthermore, user perceptions of online health information quality positively mediate the relationships.

### Social Divides Influencing Functional and Critical eHealth Literacy

Above all, our results reveal how Chinese netizens’ sociodemographic background and personal health status are manifested in individuals’ varying levels of health information efficacy. In line with previous studies [9,16], gender differences, education gaps, and income inequality are widely observed among Chinese internet users’ eHealth literacy [36,57,58]. Notably, our results showed that the younger generation is not necessarily more capable of obtaining and effectively using online health information tailored to their specific needs compared with previous studies [16,21]. Moreover, the results indicated that subhealthy groups tend to have higher levels of functional and critical literacy. Our findings validated established eHealth use models [59,60] in the Chinese contexts, demonstrating that socioeconomic divides in relation to age, education, and income, together with individual health status, are further factored into people’s actual engagement with eHealth and final health outcomes. Using post hoc pairwise comparisons, we revealed the difference in main effects on OHIS and health behaviors between various user groups. Consistent with previous research [6], older, moderately high-income, and more well-educated groups show a significantly higher engagement with OHIS and health maintenance than their corresponding counterparts.

The findings also highlighted how eHealth literacy served as a crucial enabling factor on an individual level. High functional literacy would facilitate users’ OHIS by leveraging eHealth resources; thus, users might be more likely to adopt health-promoting behaviors [61,62]. Furthermore, better critical literacy might contribute to better well-being and engagement in health behaviors [38,39], as it can increase levels of self-engagement, initiative, and control over health concerns and self-care management [19]. It is particularly important to individuals for mitigating the effect of online misinformation, especially during public health crises, such as the COVID-19 pandemic, when health misinformation is widespread [63]. Notably, we found that people who are dealing with chronic diseases are more inclined to search for professional medical information through the internet, nevertheless with relatively low eHealth literacy. This observation emphasizes the special needs of the vulnerable demographic, who are particularly sensitive to heightened health risks and susceptible to encountering low-quality eHealth content.

### OHIS Contributing to Both eHealth Literacy and Health Behaviors

Our structural model complements existing theoretical frameworks with regard to digital health literacy and OHIS, revealing the reciprocal associations existing between both functional or critical literacy and users’ OHIS. People who are more confident in navigating online resources are more inspired to devote sufficient effort to seek and use health resources in effective ways [64]. Furthermore, users’ ability to evaluate and apply health information could be better achieved by their actual engagement with the online environment [30], while frequency and diversity of internet use could also contribute to higher eHealth literacy [65]. While individuals with limited eHealth literacy might encounter more perceived barriers in using accurate health information [66], frequent OHIS might contribute to wider access to different health information where individuals can evaluate, compare, and decide how it will facilitate their health decision-making and consequently build individual eHealth literacy [67,68]. Therefore, we underscore the importance of improving general users’ functional and critical literacy, and the benefit will be amplified with more OHIS.

### Intermediary Roles of User Perceptions of Online Health Information

Last but not least, the way users perceive their ability to access and assess health information through the internet significantly influences their attitudes toward health content. Despite concerns raised by previous studies about cognitive biases in highly literate individuals favoring health information aligning with their existing beliefs, particularly regarding vaccinations [69], our results suggest that highly literate users tend to view general health information when not viewpoint-specific as more accurate, objective, relevant, applicable, and skillfully navigating around low-quality content. The quality assessment of health information affects their willingness to integrate such information into their daily lives [40,70]. For instance, high-quality, well-designed health information websites can foster positive attitudes toward OHIS, offering relevant and effective content that boosts health knowledge and outcomes [40,43]. Conversely, misleading or inaccurate information can detract from OHIS’s effectiveness [71].

### Limitations

There are limitations in our study that point to future prospects of research. First, our sampling approach may not fully represent the regional, cultural, and economic diversity of the Chinese internet user base. Second, our reliance on self-reported survey data to measure eHealth literacy and health status, despite efforts to encompass its complex nature, may not be as reliable as qualitative observations or experimental measures used in other studies [44,71,72]. Third, the cross-sectional design inhibits our ability to draw causal relationships between variables and is subject to social desirability bias. Future research could benefit from longitudinal, experimental, or observational studies to address these limitations [62]. Finally, the influence of some other sociopsychological determinants, such as perceived health threat and health anxiety, in influencing OHIS and health outcomes needs to be further examined [73]. Integrating these determinants into future studies could offer a more rounded understanding of how OHIS affects health outcomes.

### Theoretical and Practical Implications

Despite the abovementioned limitations, our study shifts the scholarly focus of OHIS from patient-centered health communication to the well-being of the general population. We presented a structural model that has expanded existing theoretical frameworks of eHealth use for health promotion and revealed the pathways linking sociodemographic determinants to eHealth literacy, the reciprocal associations between eHealth literacy and OHIS, the mediating mechanisms of perceived information quality, and how they ultimately contribute to health outcomes. Crucially, our study highlights the importance of user abilities to locate relevant and high-quality information sources in influencing health wellness [3,74]. While the lack of a precise definition and shared understanding of eHealth literacy may have impeded the progress of eHealth studies [75], we suggest that a nuanced framework of eHealth literacy could encourage more professional discussions and theory-driven research. Moreover, while prior research often focused on health literacy from a public health standpoint, our study bridges interdisciplinary discussion from public health, communication, as well as media psychology. We examined the mechanisms linking macrolevel social dynamics and cognitive processing of online health information. The potential implications for reducing health disparities linked to socioeconomic factors could be extrapolated to diverse populations and cultural contexts, thus broadening the applicability of our research, especially in the context of non–high-income countries [59].

In practical terms, our study offers actionable insights for policy makers and health practitioners aiming to devise targeted health promotion interventions for the wider public. These efforts should not only focus on enhancing the quality of online health information from the supply side but also aim to shift user attitudes, increase the experience of eHealth use, and foster health behaviors. This is especially important for individuals with limited eHealth literacy, who are at greater risk of encountering poor-quality eHealth information and facing health disparities. In addition, we emphasize the necessity of integrating critical eHealth literacy into health promotion initiatives. The design, implementation, and evaluation of health education and promotion should consider the intricacy between individuals’ orientation toward health information, their use of eHealth resources, and the broader structural disparities identified in our research framework. Particularly in non–high-income countries such as China, well-structured information campaigns are pivotal in reducing inequalities in literacy and enhancing the accessibility and utility of health information sources. Our results suggest a need for improvements that cater to the diverse literacy levels within the population, indicating a path forward for reducing health disparities and fostering equitable health outcomes.

## Appendix

Multimedia Appendix 1 Checklist for Reporting Results of Internet e-Surveys (CHERRIES).

Multimedia Appendix 2 Survey questionnaire in English and Chinese.

Multimedia Appendix 3 Descriptive statistics for participants’ eHealth literacy.

## Abbreviations

AVE: average variance extracted
CFA: confirmatory factor analysis
CFI: comparative fit index
RMSEA: root mean square error of approximation
SEM: structural equation modeling
SRMR: standardized root mean square residual
OHIS: online health information seeking

## Data Availability

The datasets generated during and analyzed during this study are available from the corresponding author on reasonable request.
