# Supplementary material for: Online Health Information Seeking, eHealth Literacy, and Health Behaviors Among Chinese Internet Users: Cross-Sectional Survey Study
Source: J Med Internet Res. 2024 Oct 18;26:e54135. doi: 10.2196/54135 (PMC11530723; doi:10.2196/54135)
Supplement: Multimedia Appendix 1 [file jmir_v26i1e54135_app1.docx]

**Multimedia Appendix 1: Checklist for Reporting Results of Internet E-Surveys (CHERRIES)**

| ***Checklist Item*** | ***Survey Details*** |
| --- | --- |
| **Describe survey design** | Cross-sectional survey (N=10000). Target population: Chinese internet users who use the internet for health information. We conducted quota sampling based on Chinese internet users’ sex, age groups, and residence according to the 48th Statistical Report on Internet Development of China (SRIDC). |
| **IRB approval** | The research protocol of this study was reviewed and received approval from the School of Humanities at Tsinghua University and the Chinese Academy of Cyberspace Studies. Additionally, the professional survey company contracted to conduct the survey executed its responsibilities in strict adherence to established research ethics. |
| **Informed consent** | At the beginning of the survey, participants were presented with information about the purpose and procedures of the study, as well as how the data would be handled. They were only allowed to proceed with the study after reading this information and providing informed consent. |
| **Data protection** | The study collected participants’ demographic information including sex, age groups, income, residence, and health status. All data was anonymized and analyzed on an aggregated level. All collected data was immediately anonymized, removing any identifiers that could directly or indirectly link the data to individual participants. Name and address were not collected. Our data collection and storage protocols were rigorously designed to be in full compliance with the Personal Information Protection Law (PIPL) of China. The anonymized data was securely stored in encrypted databases with access strictly limited to authorized research team members. |
| **Development and testing** | The survey was developed with input from media, communication, and public health experts, ensuring content relevance and clarity. The electronic questionnaire, programmed by our survey provider, underwent comprehensive usability and technical functionality tests by our research team prior to distribution, guaranteeing a smooth experience for respondents. |
| **Open survey versus closed survey** | We conducted a closed survey accessible exclusively to registered panelists of the online survey platform IDiaoYan, ensuring controlled participation. |
| **Contact mode** | The initial contact was made on the internet. |
| **Advertising the survey** | The survey announcement was disseminated via the IDiaoYan sample panel and initially reached 96,335 registered users of IDiaoYan. |
| **Web/E-mail** | The survey was posted on a website. |
| **Context** | The survey was hosted and distributed via a professional survey company in China - IDiaoYan platform, whose panel size exceeded 10million in 2019, indicative of its broad reach and varied audience base. Our study reached 96,335 of its registered users.  IDiaoYan typically attracts users interested in participating in market research surveys across different topics. This wide-ranging interest among its users ensures a diverse sample, although the platform’s general orientation towards market research may still influence the type of individuals who choose to participate, potentially impacting the survey results. For instance, users already inclined towards engaging with surveys may have specific characteristics or interests that differ from the general population. While 16,774 panel users clicked into the survey, the screening question in our survey further refined the participant pool to those meeting specific criteria. |
| **Mandatory/voluntary** | Participation was on a voluntary basis. |
| **Incentives** | Participants providing valid responses in our survey were compensated with 5,000 points (valued at RMB5) within the IDiaoYan online reward system as an incentive. |
| **Time/Date** | Data was collected between 1 November 2021 to 26 November 2021. |
| **Randomization of items or questionnaires** | No items were randomized or alternated. |
| **Adaptive questioning** | Our survey utilized adaptive questioning to streamline the experience. The initial question (Q1) identified whether respondents used the internet for health information. Affirmative responses led to Q2, which probed into their reasons for using the internet. Negative responses were routed to Q3, asking for reasons against using the internet. Respondents not engaging with online health information (N=4637) concluded their participation at this point, thus tailoring the survey’s complexity to relevant user experiences. |
| **Number of Items** | Overall the questionnaire had 41 items (including pre-screening questions and scales). |
| **Number of screens (pages)** | The survey had 7 online pages. |
| **Completeness check** | The survey implemented post-submission completeness checks to ensure all mandatory items were addressed. Of the 16,744 respondents who began the survey, 891 did not complete it. For the majority of the items, we included an 'others' open-ended option to accommodate diverse responses, and we required participants to select at least one response to proceed. |
| **Review step** | Respondents had the option to withdraw from the study at any point during the survey. However, to preserve the authenticity of responses, the survey was designed to prevent participants from revisiting and altering their answers. |
| **Unique site visitor** | A unique visitor was defined based on both IP and cookie controls, as well as device identification. |
| **View rate (Ratio of unique survey visitors/unique site visitors)** | The survey was distributed to 96,335 unique site visitors of IDiaoYan, among which 16,774 clicked into the first page of the survey. View rate is 17.4%. |
| **Participation rate (Ratio of unique visitors who agreed to participate/unique first survey page visitors)** | Among the 16,774 users who accessed the first page of the survey, all agreed to participate. However, 1,015 of these participants were unable to submit responses due to quota sampling limitations, where response collection ceases once the quota for a specific group is reached. This results in a recruitment rate of 93.95%. |
| **Completion rate (Ratio of users who finished the survey/users who agreed to participate)** | Out of the 16,774 participants who agreed to participate, 4,637 were screened out after Question 1 and not included in final analysis. Among the 12137 responses, 891 participants did not finish the survey, and 1,015 responses were not completed due to quota sampling. Consequently, 10,231 participants submitted the last questionnaire page, resulting in a completion rate of approximately 84.3%. |
| **Cookies used** | Cookies were employed to assign a unique identifier to each participant's computer. Participants were made aware of the cookie usage as part of the registration and onboarding process on the IDiaoYan platform. The survey itself did not explicitly reiterate the cookie policy. To maintain data integrity, we ensured that duplicate entries were minimized by restricting access to the survey—participants could not complete the survey more than once. |
| **IP check** | IP address was also used to identify duplicate entries. No two entries from the same IP address were allowed within 24 hours. |
| **Log file analysis** | We also authenticate respondents through comparing the filled-in demographic information with the registered profile information. |
| **Registration** | Users need to login to their IDiaoYan account to participate in the study, and the survey was not displayed a second time once the user had filled it in. |
| **Handling of incomplete questionnaires** | Only complete and valid questionnaires were analyzed (N=10000). The initial question (Q1) identified whether respondents used the internet for health information. Negative responses were routed to Q3, asking for reasons against using the internet. Respondents not engaging with online health information (N=4637) terminated their survey early and not included in the analysis. |
| **Questionnaires submitted with an atypical timestamp** | The average time for all users to complete the survey is 7.5 minutes, and we consider responses completed shorter than 3 minutes as invalid responses. |
| **Statistical correction** | N/A |
