# Supplementary material for: Online Health Information Seeking, eHealth Literacy, and Health Behaviors Among Chinese Internet Users: Cross-Sectional Survey Study
Source: J Med Internet Res. 2024 Oct 18;26:e54135. doi: 10.2196/54135 (PMC11530723; doi:10.2196/54135)
Supplement: Multimedia Appendix 2 [file jmir_v26i1e54135_app2.docx]

**Multimedia Appendix 2: Survey Questionnaire in English and Chinese**

Thank you for considering participation in our study. Our research team is conducting this survey to better understand how Chinese internet users utilize the Internet for online health information and its influence on health behaviors. Please read through this information prior to your participation.

Your participation is voluntary, and all personal information will be kept anonymous and confidential. Participants under 18 should only participate in the survey under parental guidance. None of the collected data will be associated with your personal information, and the data collected will be used for academic purpose only. If you decide to take part, you may withdraw at any point before submitting your answers by pressing the “Exit” button or closing the browser. It will take approximately 10 minutes to complete the questionnaire. You will receive 5,000 points upon completing the entire survey.

If you have read the information above and agree to participate with the understanding that the data you submit will be processed accordingly, please click the “next” button below to start. Thank you for your support.

**----Page Break----**

**Pre-screening Question**

Have you ever used the Internet to obtain health information?

1. Yes [TO Q2]
2. No [TO Q3]

Q2 What are the reasons that you use the Internet to obtain health information? (Multiple choice, up to three) [TO Q4]

1. Large volume
2. Easy to operate
3. Anonymity
4. Lower cost for accessing relevant information
5. Others___

Q3 What are the reasons that you are not using the Internet to obtain health information? (Multiple choice, up to three) [TERMINATE HERE]

1. Having difficulties accessing the Internet and mobile devices
2. Don’t have health-related concerns
3. Don’t know how to find relevant and useful health information
4. Mixed quality of the information across the Internet
5. Others___

**----Page Break----**

**Online Health Information Seeking (OHIS)**

How often do you obtain health information from the following sources? Please select the frequency that best represents your usage, with higher numbers indicating more frequent usage. (1= Never, 5=Always)

1. Mainstream state media’s (e.g., Xinhua News, People’s Daily) websites, apps, and social media accounts
2. Professional health platforms’ (e.g. DXY.COM, Chunyuyisheng, Wen Yi) websites, apps and social media accounts
3. Individual social media accounts (e.g., KOLs on Weibo, Douyin or Kuaishou)
4. Aggregated news platforms’ (e.g., Tencent News, Toutiao, NetEase News) websites and mobile apps
5. Health sectors on web portals (e.g., Sina Health, Sohu Health) and their websites, APPs and social media accounts
6. Search engine (e.g., Baidu, Sogou, Bing)
7. Social networking and Q&A forums (e.g., Zhihu, Douban)
8. Online support forums (e.g., BBS.TNBZ.COM for diabetes patients)

**----Page Break----**

**Credibility of Information Sources**

From the list of health information sources below, please rank the top three sources you consider to be the most credible.

1. Mainstream state media’s (e.g., Xinhua News, People’s Daily) websites, apps, and social media accounts
2. Professional health platforms’ (e.g. DXY.COM, Chunyuyisheng, Wen Yi) websites, apps and social media accounts
3. Individual social media accounts (e.g., KOLs on Weibo, Douyin or Kuaishou)
4. Aggregated news platforms’ (e.g., Tencent News, Toutiao, NetEase News) websites and mobile apps
5. Health sectors on web portals (e.g., Sina Health, Sohu Health) and their websites, APPs and social media accounts
6. Search engine (e.g., Baidu, Sogou, Bing)
7. Social networking and Q&A forums (e.g., Zhihu, Douban)
8. Online support forums (e.g., BBS.TNBZ.COM for diabetes patients)

**Perceptions of the Quality of Online Health Information**

To what extent do you agree with the following statements? (1= Strongly disagree; 5= Strongly agree)

1. The content of online health information is supported by reliable scientific evidence.
2. Online health information aligns with the latest advancements and consensus within the medical science community.
3. The sources of online health information are credible and reliable.
4. Accessing online health information does not pose a risk to my personal health and well-being.
5. The advice contained in online health information is actionable and applicable in real-life scenarios.

**----Page Break----**

**E-Health Literacy**

To what extent do the following statements reflect your personal situation? (1 = Not at all; 5 = Extremely well)

1. I know how to find helpful health resources on the Internet (FL)
2. I know what health resources are available on the Internet (FL)
3. I have the skills I need to evaluate the health resources I find on the Internet (CL)
4. I can tell high quality from low quality health resources on the Internet (CL)
5. I am capable of using mobile devices (e.g., smartphones, tablets) to search for health information online. (FL)
6. I can distinguish between different sources of health information, such as authoritative sources and primary sources (e.g., medical records) (CL)
7. I can effectively use relevant keywords and logical search operators when querying or retrieving health information online (FL)
8. I have the skills to open and navigate different web pages and websites to access health information across the internet (FL)
9. I know how to bookmark or save useful health information from online sources (FL)
10. I feel confident in using information from the Internet to make health decisions (CL)

FL: Functional literacy; CL: Critical literacy

**----Page Break----**

**Health Maintenance Behavior**

To what extent do the following statements suit your own situation? (1= Not at all; 5=Extremely well)

1. My daily meals are mainly cereals, with a good mix of meat and vegetables, and a good mix of coarse and fine foods
2. I maintain a healthy weight by being physically active, less sedentary and more active every day
3. I value and maintain my mental health and seek help when I encounter mental problems
4. I wash my hands regularly, bathe frequently, brush my teeth in the morning and evening, rinse my mouth after meals, and do not share towels and toiletries

**----Page Break----**

What is your biological sex?

1. Female
2. Male

Which age group do you belong to?

1. Under 19
2. 20-29
3. 30-39
4. 40-49
5. 50-59
6. Above 60

The province you reside in is _______

Do you reside in urban or rural areas?

1. Urban
2. Rural

What is your monthly income level?

1. Under￥1,500
2. ￥1,500 - 3,000
3. ￥3,001 - 5,000
4. ￥5,001 – 8,000
5. ￥8,001 - 12,000
6. ￥12,001 – 20,000
7. ￥20,000 above

What is the highest educational level you have achieved?

1. Primary school or less
2. Middle school
3. High school or secondary vocational school
4. Associate degree
5. Bachelor degree
6. Master and above

What is your occupation?

1. Student
2. Government institution staff
3. Manager of an enterprise/company
4. Professional and technical personnel
5. Business service worker
6. Manufacturing production enterprises
7. Self-employed/freelance workers
8. Rural migrant workers
9. Agricultural, forestry and fishery laborer
10. Retirees
11. Property/lay-off/unemployed persons
12. Others ____

Which one do you think better describe your current health status?

1. Suffering from a severe disease (such as cancer, disability, etc.)
2. Suffering from chronic diseases (like hypertension, coronary heart disease, diabetes, arthritis, etc.)
3. Sub-health (such as fatigue, poor sleep, poor appetite, dizziness, forgetfulness, etc.)
4. Not bad (easy to get sick and the immunity is low)
5. Good (eat and sleep normally, full of energy, etc.)

您好！感谢您对本研究的兴趣。为了全面了解中国网民网络健康信息需求、使用习惯与网络健康素养情况，本课题组展开此项问卷调查。请在认真阅读以下说明后决定是否继续作答。

参与此项研究基于自愿原则，您的所有回答及个人信息都将被匿名化处理，同时严格保密。18岁以下的未成年人需在监护人陪同下作答。所有回答将仅作为学术研究使用，且不会涉及到个人隐私。在开始作答后，您随时可以选择终止此项研究并“退出”问卷页面。完成作答后您将获得5000积分奖励。完成此问卷大致需要10分钟。

如果您已认真阅读以上说明，且对研究过程及数据处理方式无异议，请点击“下一页”开始答题。感谢您的支持。

-------

您是否曾使用互联网获取健康信息？

1. 会 (至第二题)
2. 不会 (至第三题)

Q2 您使用互联网获取健康信息的主要原因为？（多选，最多选3项）

1. 信息量大
2. 操作简单便捷
3. 网络具有匿名性
4. 信息获取成本低
5. 其他______

Q3 您不使用互联网获取健康信息的主要原因为？（多选，最多选3项）

1. 难以接触网络和移动设备
2. 没有健康相关困扰
3. 不知道如何找到有用的健康信息
4. 网络健康信息质量鱼龙混杂
5. 其他________

**[注：不会通过互联网获取健康信息的答题者在此处终止作答]**

----

您通过以下各类网络渠道获取健康信息的频率为？

| 网络信息渠道 | 从不 | 很少 | 有时 | 经常 | 总是 |
| --- | --- | --- | --- | --- | --- |
| 权威类媒体（如新华社、人民日报）的网站、客户端、微信微博等社媒账号 | 1 | 2 | 3 | 4 | 5 |
| 专业类健康平台（如丁香医生、春雨、问医网）的网站、客户端、微信微博等社媒账号 | 1 | 2 | 3 | 4 | 5 |
| 公共社媒平台的自媒体账号（如微博健康博主、抖音或快手平台大V） | 1 | 2 | 3 | 4 | 5 |
| 聚合类资讯平台（如腾讯新闻、今日头条、网易新闻）的网站、客户端 | 1 | 2 | 3 | 4 | 5 |
| 门户平台健康版块（如新浪健康、搜狐健康）的网站、客户端、社媒账号 | 1 | 2 | 3 | 4 | 5 |
| 搜索引擎（如百度、搜狗、必应）的网页版、客户端 | 1 | 2 | 3 | 4 | 5 |
| 综合问答类网站/论坛（如知乎、豆瓣） | 1 | 2 | 3 | 4 | 5 |
| 健康类在线互助论坛（如“甜蜜家园”糖尿病论坛） | 1 | 2 | 3 | 4 | 5 |

----

请您为以下不同网络信息渠道的权威度排序（排出前3名即可）

| 网络信息渠道 |
| --- |
| 权威类媒体（如新华社、人民日报）的网站、客户端、微信微博等社媒账号 |
| 专业类健康平台（如丁香医生、春雨、问医网）的网站、客户端、微信微博等社媒账号 |
| 公共社媒平台的自媒体账号（如微博健康博主、抖音或快手平台大V） |
| 聚合类资讯平台（如腾讯新闻、今日头条、网易新闻）的网站、客户端 |
| 门户平台健康版块（如新浪健康、搜狐健康）的网站、客户端、社媒账号 |
| 搜索引擎（如百度、搜狗、必应）的网页版、客户端 |
| 综合问答类网站/论坛（如知乎、豆瓣） |
| 健康类在线互助论坛（如“甜蜜家园”糖尿病论坛） |

您在多大程度上认同以下论述？

| 论述 | 非常不认同 | 较不认同 | 中立 | 较为认同 | 非常认同 |
| --- | --- | --- | --- | --- | --- |
| 网络平台的健康信息内容有可靠的科学证据 | 1 | 2 | 3 | 4 | 5 |
| 网络平台的健康信息符合医学科学领域最新的进展和共识 | 1 | 2 | 3 | 4 | 5 |
| 网络平台的健康信息来源是可以信赖的 | 1 | 2 | 3 | 4 | 5 |
| 网络平台的健康信息不会危害到我的健康利益 | 1 | 2 | 3 | 4 | 5 |
| 网络平台的健康信息内容可付诸实践 | 1 | 2 | 3 | 4 | 5 |

------

以下论述在多大程度上符合您的实际情况？

| 论述 | 非常不符合 | 较不符合 | 一般 | 较为符合 | 非常符合 |
| --- | --- | --- | --- | --- | --- |
| 我知道如何上网查找有用的健康信息资源 | 1 | 2 | 3 | 4 | 5 |
| 我知道从网络上可以获取哪些类型的健康资源信息 | 1 | 2 | 3 | 4 | 5 |
| 我具备评价网络健康资源信息好坏的能力 | 1 | 2 | 3 | 4 | 5 |
| 我能够区分网络上高质量和低质量的健康资源信息 | 1 | 2 | 3 | 4 | 5 |
| 我能熟练运用网络移动设备（如手机）查找健康信息 | 1 | 2 | 3 | 4 | 5 |
| 我能够区分权威资料、原始资料（如体检信息）等不同信息来源之间的区别 | 1 | 2 | 3 | 4 | 5 |
| 我能有效利用关键词和逻辑连接词检索健康信息 | 1 | 2 | 3 | 4 | 5 |
| 我能熟练地打开和浏览不同网页的健康信息内容 | 1 | 2 | 3 | 4 | 5 |
| 我知道如何收藏或保存有用的健康信息内容 | 1 | 2 | 3 | 4 | 5 |
| 我对利用网络获取的信息作出健康决定充满信心 | 1 | 2 | 3 | 4 | 5 |

------

以下论述在多大程度上符合您的实际情况？

| 论述 | 非常不符合 | 较不符合 | 一般 | 较为符合 | 非常符合 |
| --- | --- | --- | --- | --- | --- |
| 我的日常膳食以谷类为主，能够注意荤素、粗细搭配 | 1 | 2 | 3 | 4 | 5 |
| 我每日会进行适量的身体活动，少静多动，减少久坐，保持健康体重 | 1 | 2 | 3 | 4 | 5 |
| 我重视和维护自身心理健康，遇到心理问题时主动寻求帮助 | 1 | 2 | 3 | 4 | 5 |
| 我能做到勤洗手、常洗澡、早晚刷牙、饭后漱口，不共用毛巾和洗漱用品 | 1 | 2 | 3 | 4 | 5 |

------

您的性别为？

1. 女性
2. 男性

您的年龄为？

1. 19岁及以下
2. 20-29岁
3. 30-39岁
4. 40-49岁
5. 50-59岁
6. 60岁及以上

您所在的省份为？下拉选项

您的居住地为？

1. 城市
2. 农村

您的月收入水平为？

1. 1500元以下
2. 1500-3000元
3. 3001-5000元
4. 5001-8000元
5. 8000-12000元
6. 12000-20000元
7. 20000元以上

您的最高受教育水平为？

1. 小学及以下
2. 初中
3. 高中/中专/技校
4. 大学专科
5. 大学本科
6. 研究生及以上

您的职业为？

1. 学生
2. 党政机关事业单位工作人员
3. 企业/公司管理人员
4. 专业技术人员
5. 商业服务业人员
6. 制造生产型企业人员
7. 个体户/自由职业者
8. 农村外出务工人员
9. 农林牧渔劳动人员
10. 退休人员
11. 物业/下岗/失业人员
12. 其他____

您的健康状况为？

1. 患有严重疾病（如癌症、残疾等）
2. 患有慢性疾病（如高血压、冠心病、糖尿病、关节炎等）
3. 亚健康（如疲劳、睡眠不佳、食欲不振、头晕、健忘等）
4. 健康状况一般（容易生病，免疫力低）
5. 健康状况良好（吃睡正常，精力充沛等）
