# Supplementary material for: Online Health Information Seeking, eHealth Literacy, and Health Behaviors Among Chinese Internet Users: Cross-Sectional Survey Study
Source: J Med Internet Res. 2024 Oct 18;26:e54135. doi: 10.2196/54135 (PMC11530723; doi:10.2196/54135)
Supplement: Multimedia Appendix 3 [file jmir_v26i1e54135_app3.docx]

**Multimedia Appendix 3: Descriptive statistics for participants’ eHealth literacy**

Table S1: Participants’ demographic characteristics and eHealth literacy (N=10,000).

| Demographics | | Participants, n (%) | Functional literacy | | Critical literacy | |
| --- | --- | --- | --- | --- | --- | --- |
|  | |  | Mean (SD) | Median (IQR) | Mean (SD) | Median (IQR) |
| **Sex** | |  |  |  |  |  |
|  | Female, n (%) | 4880 (48.8) | 3.86 (0.62) | 3.83 (3.50-4.33) | 3.55 (0.69) | 3.50 (3.25-4.00) |
|  | Male, n (%) | 5120 (51.2) | 3.82 (0.64) | 3.83 (3.50-4.33) | 3.56 (0.67) | 3.50 (3.00-4.00) |
| **Age group (years)** | | |  |  |  |  |
|  | <19 | 1560 (15.6) | 3.81 (0.60) | 3.83 (3.50-4.33) | 3.45 (0.66) | 3.50 (3.00-4.00) |
|  | 20 to 29 | 1740 (17.4) | 3.90 (0.59) | 4.00 (3.50-4.33) | 3.59 (0.65) | 3.50 (3.20-4.00) |
|  | 30 to 39 | 2030 (20.3) | 3.96 (0.55) | 4.00 (3.67-4.33) | 3.64 (0.65) | 3.75 (3.25-4.25) |
|  | 40 to 49 | 1870 (18.7) | 3.91 (0.59) | 4.00 (3.50-4.33) | 3.62 (0.65) | 3.75 (3.25-4.00) |
|  | 50 to 59 | 1590 (15.9) | 3.76 (0.66) | 3.83 (3.33-4.33) | 3.54 (0.70) | 3.50 (3.00-4.00) |
|  | >60 | 1210 (12.1) | 3.59 (0.75) | 3.67 (3.17-4.17) | 3.42 (0.78) | 3.50 (3.00-4.00) |
| **Residential area** | | |  |  |  |  |
|  | Urban | 7060 (70.6) | 3.89 (0.61) | 4.00 (3.50-4.33) | 3.57 (0.67) | 3.50 (3.25-4.00) |
|  | Rural | 2940 (29.4) | 3.73 (0.67) | 3.83 (3.33-4.33) | 3.51 (0.70) | 3.50 (3.00-4.00) |
| **Education level** | | |  |  |  |  |
|  | Primary school or less | 174 (1.7) | 3.44 (0.72) | 3.50 (3.00-4.00) | 3.34 (0.78) | 3.50 (2.75-4.00) |
|  | Middle school | 1066 (10.7) | 3.71 (0.67) | 3.83 (3.33-4.17) | 3.46 (0.71) | 3.50 (3.00-4.00) |
|  | High school or secondary vocational school | 2499 (25) | 3.76 (0.67) | 3.83 (3.33-4.33) | 3.51 (0.69) | 3.50 (3.00-4.00) |
|  | Associate degree | 2588 (25.9) | 3.87 (0.60) | 3.83 (3.50-4.33) | 3.57 (0.67) | 3.50 (3.25-4.00) |
|  | Bachelor degree | 3393 (33.9) | 3.93 (0.58) | 4.00 (3.50-4.33) | 3.62 (0.66) | 3.75 (3.25-4.00) |
|  | Master and above | 280 (2.8) | 3.90 (0.62) | 4.00 (3.50-4.33) | 3.56 (0.67) | 3.50 (3.25-4.00) |
| **Income levelb** | |  |  |  |  |  |
|  | <￥1500 | 1267 (12.7) | 3.76 (0.64) | 3.83 (3.33-4.17) | 3.41 (0.69) | 3.50 (3.00-4.00) |
|  | ￥1500 to 3000 | 1286 (12.9) | 3.73 (0.68) | 3.83 (3.33-4.33) | 3.41 (0.72) | 3.50 (3.00-4.00) |
|  | ￥3001 to 5000 | 2294 (22.9) | 3.80 (0.65) | 3.83 (3.50-4.33) | 3.51 (0.68) | 3.50 (3.00-4.00) |
|  | ￥5001 to 8000 | 2632 (26.3) | 3.88 (0.60) | 3.83 (3.50-4.33) | 3.61 (0.66) | 3.75 (3.25-4.00) |
|  | ￥8001 to 12,000 | 1629 (16.3) | 3.94 (0.59) | 4.00 (3.50-4.50) | 3.68 (0.65) | 3.75 (3.25-4.25) |
|  | ￥12,001 to 20,000 | 693 (6.9%) | 3.92 (0.60) | 4.00 (3.50-4.33) | 3.68 (0.65) | 3.75 (3.25-4.25) |
|  | >￥20,000 | 199 (2) | 3.95 (0.58) | 4.00 (3.50-4.33) | 3.73 (0.66) | 3.75 (3.25-4.25) |
| **Health status** | |  |  |  |  |  |
|  | Experiencing a severe disease | 46 (0.5) | 3.54 (0.90) | 3.58 (2.96-4.33) | 3.37 (0.73) | 3.50 (3.00-3.75) |
|  | Experiencing chronic diseases | 1397 (14) | 3.70 (0.70) | 3.83 (3.33-4.33) | 3.50 (0.73) | 3.50 (3.00-4.00) |
|  | Subhealth symptoms | 2661 (26.6) | 3.88 (0.61) | 3.83 (3.50-4.33) | 3.59 (0.68) | 3.50 (3.25-4.00) |
|  | Not bad | 2119 (21.2) | 3.82 (0.62) | 3.83 (3.50-4.33) | 3.53 (0.69) | 3.50 (3.00-4.00) |
|  | Good | 3777 (37.8) | 3.88 (0.61) | 3.83 (3.50-4.33) | 3.57 (0.66) | 3.50 (3.25-4.00) |
| **Region** | |  |  |  |  |  |
|  | East | 5628 (56.3) | 3.85 (0.63) | 3.83 (3.50-4.33) | 3.57 (0.68) | 3.50 (3.25-4.00) |
|  | Central | 1998 (20) | 3.83 (0.63) | 3.83 (3.50-4.33) | 3.51 (0.70) | 3.50 (3.00-4.00) |
|  | West | 1630 (16.3) | 3.82 (0.64) | 3.83 (3.50-4.33) | 3.49 (0.69) | 3.50 (3.00-4.00) |
|  | Northeast | 744 (7.4) | 3.87 (0.60) | 3.83 (3.50-4.33) | 3.67 (0.65) | 3.75 (3.25-4.25) |

^a^Test statistics were reported for dichotomous variables (*z* score) and multicategorical variables (*χ^2^*) alongside *P* value.

^b^Conversion rate at the time of the study: 1CNY=0.16USD

Table S2: Distribution of participants across 31 provinces.

| **Province** | **Percent** | **Region** | **Functional literacy, Median(IQR)** | **Critical Literacy, Median(IQR)** |
| --- | --- | --- | --- | --- |
| Beijing | 7.91% | East | 3.5 (4-4.33) | 3.2 (3.8-4.2) |
| Shanghai | 6.83% | East | 3.5 (3.83-4.33) | 3.2 (3.6-4.2) |
| Guangdong | 13.86% | East | 3.5 (3.83-4.33) | 3.2 (3.6-4) |
| Tianjin | 2.60% | East | 3.5 (3.83-4.33) | 3.25 (3.8-4.2) |
| Hebei | 6.07% | East | 3.5 (3.83-4.33) | 3.2 (3.6-4) |
| Shanxi | 3.50% | Middle | 3.5 (3.83-4.33) | 3.2 (3.6-4) |
| Liaoning | 3.36% | Northeast | 3.5 (4-4.33) | 3.4 (3.8-4.2) |
| Jilin | 2.08% | Northeast | 3.67 (4-4.33) | 3.4 (3.8-4.2) |
| Heilongjiang | 2.00% | Northeast | 3.33 (3.83-4.33) | 3.2 (3.6-4) |
| Jiangsu | 5.64% | East | 3.5 (4-4.33) | 3.2 (3.6-4.2) |
| Zhejiang | 4.74% | East | 3.63 (3.83-4.33) | 3.2 (3.6-4) |
| Anhui | 3.16% | Middle | 3.33 (3.83-4.33) | 3.2 (3.6-4.2) |
| Fujian | 2.64% | East | 3.33 (3.83-4.33) | 3.2 (3.6-4) |
| Jiangxi | 2.17% | Middle | 3.5 (3.83-4.17) | 3.2 (3.6-4) |
| Shandong | 5.50% | East | 3.5 (3.92-4.33) | 3.2 (3.6-4.2) |
| Henan | 4.77% | Middle | 3.5 (3.83-4.33) | 3.2 (3.6-4) |
| Hubei | 3.34% | Middle | 3.5 (4-4.33) | 3.2 (3.8-4.2) |
| Hunan | 3.04% | Middle | 3.5 (3.83-4.33) | 3.2 (3.6-4) |
| Guangxi Zhuang Autonomous Region | 2.43% | West | 3.33 (3.83-4.33) | 3.2 (3.6-4) |
| Chongqing | 1.90% | West | 3.46 (4-4.33) | 3.2 (3.6-4) |
| Sichuan | 4.31% | West | 3.5 (3.83-4.33) | 3.2 (3.6-4) |
| Yunnan | 1.47% | West | 3.33 (3.83-4.17) | 3.2 (3.6-4) |
| Shaanxi | 1.95% | West | 3.5 (3.83-4.17) | 3.2 (3.6-4) |
| Inner Mongolia Autonomous Region | 0.71% | West | 3.17 (3.83-4.17) | 3 (3.6-4) |
| Hainan | 0.49% | East | 3.5 (4-4.5) | 3.2 (3.6-4.1) |
| Guizhou | 1.14% | West | 3.5 (3.83-4.33) | 3.15 (3.6-4) |
| Gansu | 0.99% | West | 3.33 (3.83-4.5) | 2.8 (3.4-4) |
| Qinghai | 0.30% | West | 3.33 (4-4.5) | 3.35 (3.8-4.2) |
| Ningxia Hui Autonomous Region | 0.40% | West | 3.5 (3.92-4.5) | 3.2 (3.5-4.2) |
| Xinjiang Uyghur Autonomous Region | 0.40% | West | 3.54 (4-4.33) | 3.05 (3.6-4.2) |
| Tibet Autonomous Region | 0.30% | West | 3.5 (4.17-4.38) | 3.35 (3.8-4.2) |
